# Increased serum amyloid A as potential diagnostic marker for lung cancer: a meta-analysis based on nine studies

**DOI:** 10.1186/s12885-016-2882-0

**Published:** 2016-11-03

**Authors:** Rong Biaoxue, Liu Hua, Gao Wenlong, Yang Shuanying

**Affiliations:** 1 Department of Respiratory Medicine, First Affiliated Hospital, Xi’an Medical University, 48 Fenghao West Road, Xi’an, 710077 China; 2 Research Center of Prevention and Treatment of Respiratory Disease, Xi’an, Shaanxi Province 710077 China; 3 Department of Respiratory Medicine, Gansu Provincial Hospital, Lanzhou, China; 4 Department of Statistics and Epidemiology, Medical College, Lanzhou University, Lanzhou, China; 5 Department of Respiratory Medicine, Second Affiliated Hospital, Xi’an Jiaotong University, Xi’an, China

**Keywords:** Serum amyloid A, SAA, Meta-analysis, Lung cancer, Diagnosis

## Abstract

**Background:**

Previous studies have disclosed that serum amyloid A (SAA) is likely involved in the lung cancer pathogenesis and progression. We performed a systematic evaluation and meta-analysis to disclose the correlation between the expression of SAA and lung cancer and to evaluate its value for lung cancer diagnosis.

**Methods:**

We searched the relevant articles from the databases of Medline, Embase, Cochrance Library and Web of Science and calculated the standardized mean difference (SMD) with 95 % confidence interval (CI) to assess the expression difference of SAA between lung cancer and normal patients. Moreover, we counted the positive rate, sensitivity and specificity and drew a summary receiver operating characteristic curve (SROC) to evaluate the diagnostic value of SAA for lung cancer.

**Results:**

A total of nine studies with 1392 individuals were included in this analysis. The results showed an increased SAA was correlated with the incidence of lung cancer (*P* < 0.001), especially with the lung squamous cell carcinoma (LSCC) (*p* = 0.012). The overall sensitivity and specificity of SAA for discerning lung cancer was 0.59 (95 % CI: 0.54–0.63) and 0.92 (95 % CI: 0.88–0.95), respectively. The area under the SROC curve was 0.9066 (SE = 0.0437).

**Conclusions:**

Increased SAA in lung cancer was intimately correlated with the development and progression of lung cancer. A higher specificity of SAA suggested that it should be a significant biomarker for discerning lung cancer from normal individuals, especially for LSCC (*p* = 0.012).

## Background

Lung cancer has become the first cause of cancer-associated death in the world [1]. This is a consistent opinion that early diagnosis and individualized therapy are conducive to improve the prognosis of lung cancer [2]. Many studies have demonstrated that abnormal protein expressions and gene mutations are correlated with the ontogenesis and progression of lung cancer [2], and reliable biomarkers derived from these abnormal molecules are more likely to help make the medical decision for individualized therapy [3]. We also know that the high mortality of lung cancer is mainly due to early metastasis and progression, and early diagnosis of lung cancer can increase the 5-year survival rate from 15 to 80 % [4]. Thus, new technology on early diagnosis and therapies are greatly required.

Recently, chronic inflammation has been showed to be associated with tumor progression, and many inflammatory factors could serve as diagnostic and prognostic biomarkers for special tumors [5]. There is common view that inflammation can become chronic processes that may promote angiogenesis and proliferation of cells, thus it may play a clear role in carcinogenesis and pathogenesis [6]. Serum amyloid A (SAA), a kind of cytokine-induced, acute inflammatory response proteins, has been known to be likely involved in cancers [7]. Research shows that liver is mainly workplace for producing SAA protein which can stimulates the production of various cytokines, and SAA plays an important role in acute immune response [8]. SAA protein in blood of patients with cancer often rises at its early stage, which have been identified both by immunochemistry and by proteomics methods in different cancers, such as lung, ovarian, renal, uterine, nasopharyngeal cancer and in melanoma [7].

Up to now, lung cancer, a very common malignant tumor, has been considered as an inflammatory disease, and the development of lung cancer correlates various cell factors and inflammatory mediators. Previous studies have specially investigated the relationship between SAA and lung cancer. These studies suggest that higher SAA can distinguish lung cancer patients from healthy individuals as well as predict the prognosis of lung cancer [9], which may be a potentially non-invasive biomarker for lung cancer. Here, we reviewed the medical literature as completely as possible, and conducted a meta-analysis to show the relationship between the expression of SAA and lung cancer and evaluate its value for lung cancer diagnosis.

## Methods

### Literature searching

The databases that we searched studies on SAA and lung cancer included Medline, Embase, Cochrance Library and Web of Science. The time scope that we defined was from the start of each database up to June 2016. The key words that we used for searching literature included: “lung cancer,” “lung malignancy,” “lung malignant tumor,” “lung neoplasms,” “serum amyloid A,” and “SAA.” We also conducted secondary searches for additional studies that regarding the SAA and lung cancer from the reference lists of included studies.

### Inclusion and exclusion criteria of literature

The inclusion criteria: (1) patients in study must be histologically diagnosed with lung cancer; (2) must be case–control or cohort association studies; (3) detection method of CAA must be able to show the continuous variables; (4) studies must have reported sufficient quantitative data; and (5) the methods of data collection and analysis must be statistically acceptable. The exclusion criteria: (1) non-original reports (such as abstracts, letters, editorials and expert opinions and case reports); (2) did not report clearly serum level of SAA with continuous variables; (3) did not contain distinctively normal control; (4) patients had been given the chemotherapy and surgery before taking blood samples; and (5) non-human studies.

### Extraction of study variables

The extracted data included: (1) authors, countries and publication date; (2) study design and case number of different groups; (3) gender and age of patients; (4) tumor node metastases (TNM) classification of lung cancer patients; (5) histological classification; (6) detection method of SAA; (7) SAA level; (8) the number of true positives, true negatives, false positives, and false negatives.

### Methodological quality assessment

We adopted the guidelines of the QUADAS-2 [10, 11] (maximum score 14) tool to assess the methodological quality of included studies, in which appraisal is performed by empirical evidence, expert opinion, and formal consensus on assessing the quality of primary studies of diagnostic accuracy [11]. In order to reduce the bias and improve the reliability, two authors independently assessed and reached a consensus. If there were a discrepancy, we would invite another expert to discuss it and reach a consistent opinion.

### Statistical analysis

We performed the statistical analysis according to the following research idea. The standardized mean difference (SMD) and their 95 % confidence intervals (CI) of lung cancer associated with the SAA was calculated directly from the data given in the eligible studies using two different meta-analysis approaches (fixed effect method and random effect method). The heterogeneity test between studies was assessed by the Chi-square test and I^2^. In the absence of heterogeneity, we used the fixed effects method, otherwise the random effect method was used. The overall effect of meta-analysis was tested using Z-scores with a significance of being set at *p* <0.05. We also ran a sensitivity analysis to determine whether the overall effect was affected by individual study. The publication bias was evaluated using Begg’s and Egger’s test respectively. Moreover, we drew a summary receiver operating characteristic (SROC) curve to determine the joint distribution of sensitivity and specificity. Statistical analysis was performed using SPSS (Version 22.0, Chicago, USA), RevMan 4.2 (Cochrane Collaboration), Meta DiSc statistical software (Version 1.4, Madrid, Spain), and Stata version 12.0 (TX, USA). All the tests were two-sided and the significant level was 0.05.

## Results

### Searching of literature

**Fig. 1:**
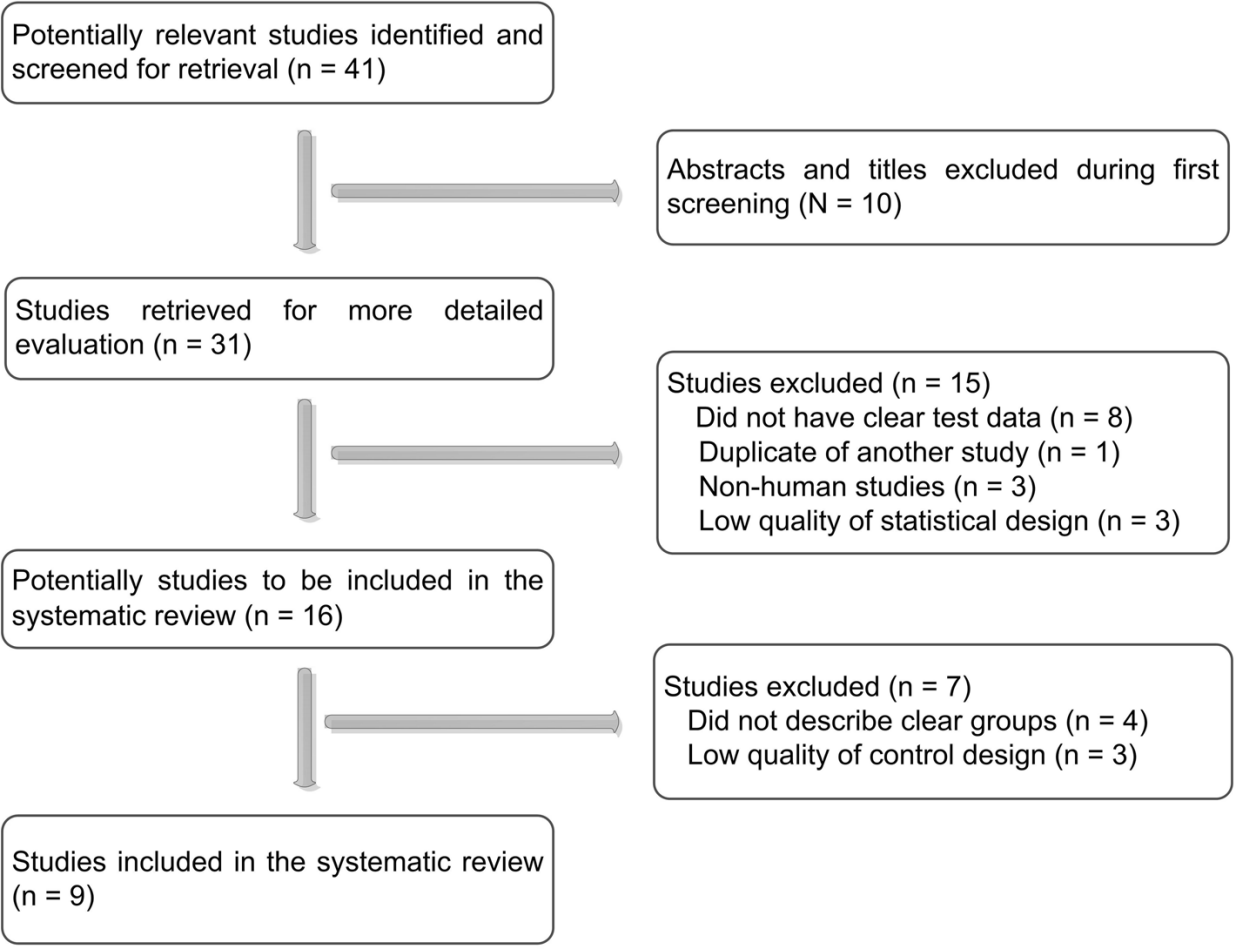
Flow chart of selection process for studies included in meta-analysis

Initially, a searching for the medical literature related to SAA and lung cancer identified 39 studies, and added two reports that were from the bibliographies of relevant articles. Of these 41 articles, 31 seemed to be eligible for the inclusion criteria. Subsequently, fifteen studies were excluded because of the following reasons: eight did not provide useful data; one was duplication of another study; three were not studies on human; and three had flaws on statistical design. However, we had to abandon seven of 16 remaining articles because they were short of clear control groups. Finally, nine publications [4, 9, 12–18] that fulfilled all of the inclusion criteria were recruited for the further analysis (Fig. 1).

### Studies description

A total of nine studies with 1392 patients included in this analysis, and ranged in study size from 34 [13] to 380 [4] patients, and ranged in age from 32 to 87 years old [14]. The studies were performed in East Asia [4, 9, 15–17], Europe [18] and America [12–14]. The histological classification of lung cancer mainly contained lung adenocarcinoma (LAC) (381 patients), lung squamous cell carcinoma (LSCC) (347 patients) and small cell lung cancer (195 patients). We established a meta-analysis database according to the extracted information (Table 1).

**Table 1:** Description of the included studies

Authors	Year	All cases (control/cancer)	Age (years)		Gender (Male/female) (N)		Histology of lung cancer (N)				Tumor Stage of lung cancer
			Control	Lung cancer	Control	Lung cancer	LAC	LSCC	SCLC	Others	
Benson MD [12]	1986	100 (50/50)	37 ± 1.9	61.1 ± 1.2	50/0	50/0	5	29	10	NA	I-IV
Howard BA [13]	2003	34 (15/19)	50.7 (23–77)		22/12		17		2	0	NA
Khan N [14]	2004	50 (25/25)	62.9 (46–83)	66.1 (47–87)	16/9	16/9	9	6	NA	8	I-IV
Dai S [16]	2007	218 (43/175)	NA	NA	NA	NA	59	78	38	0	NA
Liu DH [17]	2007	275 (82/194)	NA	65 (41–76)	NA	135/58	65	97	31	0	I-IV
Cho WC [15]	2010	189 (35/154)	NA	65.5 ± 0.9	NA	129/25	50	53	38	13	I-IV
Sung HJ [4]	2011	380 (140/240)	59 (37–84)	57 (32–79)	96/44	217/53	140	50	50	0	I–IV
Dowling P [18]	2011	109 (30/79)	56.5 ± 7	61 ± 9.3	15/15	31/24	36	17	26	0	IIIB–IV
Kanoh Y [9]	2013	37 (13/24)	62.6 (53–72)	66.2 (52–79)	NA	NA	24		0	0	I-IV

*NA* unavailable; *N* cases; *LAC* lung adenocarcinoma; *LSCC* lung squamous cell carcinoma; *SCLC* small cell lung cancer

### Study quality assessment

Tables 2 showed general information of included studies. Of these studies, five were retrospective [4, 13, 15, 16, 18], one was retrospective [12], and the other three studies did not report they were prospective or retrospective [9, 14, 17]. In addition, five studies tested the concentration of SAA using enzyme-linked immunosorbent assays (ELISA) [4, 13, 14, 16, 18], and the rest using competitive binding radioimmunoassay [12], protein chip array [17], quantitative analysis [15] and latex nephelometry [9] respectively. We assessed the quality of studies according to the QUADAS-2 scoring system. Overall, the QUADAS-2 scores of six studies was more than 10 [4, 12, 14, 15, 17, 18] and that of three less than 10 [9, 13, 16].

**Table 2:** Methodology and quality of inclined studies

Authors	Year	Research design	Country	Asians/Caucasians	Test method	Measurement units	QUADAS
Benson MD [12]	1986	Prospective	Indiana	0/100	Competitive binding radioimmunoassay	u/ml	12
Howard BA [13]	2003	Retrospective	USA	0/19	ELISA	ng/mL	7
Khan N [14]	2004	NA	North Carolina	0/50	MALDI-TOF; ELISA	ng/mL	10
Dai S [16]	2007	Retrospective	China	175/0	SELDI-TOF-MS; ELISA	Peak intensities	9
Liu DH [17]	2007	NA	China	275/0	Protein Chip Array	Peak intensities	10
Cho WC [15]	2010	Retrospective	Hong Kong	189/0	SELDI-TOF; quantitative analysis	Peak intensities; ug/mL^−1^	11
Sung HJ [4]	2011	Retrospective	South Korea	380/0	LC-ESI-MS/MS Analysis; ELISA	ug/mL	12
Dowling P [18]	2011	Retrospective	Ireland	0/109	ELISA	ug/mL	10
Kanoh Y [9]	2013	NA	Japan	37/0	Latex nephelometry	ug/mL	7

*QUADAS* quality assessment for studies of diagnostic accuracy (maximum score 14); *ELISA* enzyme-linked immunosorbent assays; *MALDI-TOF* matrix assisted laser desorption ionization time of flight; *SELDI-TOF-MS* surface-enhanced laser desorption/inionation-time of flight-mass spectra; *LC-ESI-MS/MS* liquid chromatography-electrospray ionisation-tandem mass spectrometry *NA* unavailable

### Heterogeneity test

The Chi-square value for the heterogeneity test of nine studies was 144.93 with 8° of freedom (d.f.) and *P* < 0.05, which meant the presence of heterogeneity in these studies. Subsequently, we reviewed each of included studies carefully from different aspects, and confirmed that the different detection methods of SAA contributed to the heterogeneity. However, there was a very good clinical homogeneity in intention and design of study in selected studies. It is common opinion about meta- analysis that clinical homogeneity is more crucial than data alone, and we could decrease this risk of heterogeneity through a method of subgroup analysis as much as possible. Thus, we finally used the random-effect model to perform this analysis [11].

### Comparison of SAA level between lung cancer and healthy individuals

**Fig. 2:**
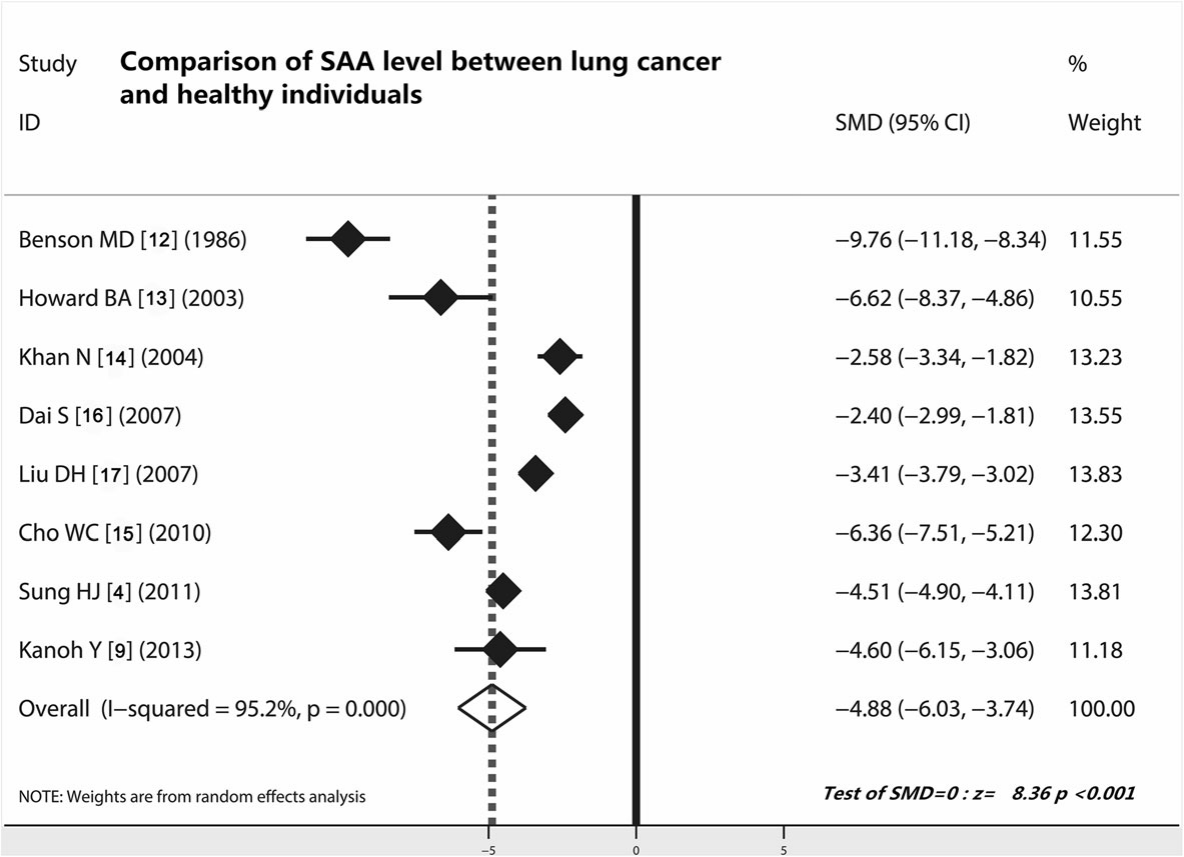
Comparison of SAA level between lung cancer patients and healthy individuals. Patients with lung cancer showed a higher SAA value than those of healthy individuals (*z* = 8.36, *P* < 0.0001); SAA, serum amyloid A; ELISA, enzyme-linked immunosorbent assays; CI, confidence interval

As shown in Table 3, eight studies compared the expression level of SAA in lung cancer and healthy group. The weight of included studies ranged from −2.40 % to −9.76 %, and the pooled SMD was −4.88 and 95 % confidence interval (CI) were −6.03 to −3.74) (Fig. 2), which indicated that patients with lung cancer had a higher SAA level than those of healthy group. The results indicated that higher SAA was a concomitant event of lung cancer (*z* = 8.36, *P* < 0.001).

**Table 3:** Data extract of SAA expression in control and cancer patients

Author	Concentration of SAA (Mean ± standard deviation)					Positive /all (N)		Diagnostic test (2 × 2 table)			
	Control	All cancer cases	LAC	LSCC	SCLC	Control	Lung cancer	TP	FP	FN	TN
Benson MD [12]	135 ± 31	1880 ± 251	1460 ± 494	1923 ± 317	1118 ± 317	14/50	45/50	45	14	5	36
Howard BA [13]	34.1	286	NA	NA	NA	1/15	15/19	15	1	4	14
Khan N [14]	43.8 ± 9.65	83.5 ± 19.5	NA	NA	NA	4/25	14/25	14	4	11	21
Dai S [16]	2.15 ± 0.73	18.48 ± 21.22	NA	NA	NA	NA	NA	NA	NA	NA	NA
Liu DH [17]	2.1 ± 0.7	19.3 ± 20.6	15.1 ± 22.4	34.3 ± 20.2	25.7 ± 26	0/82	95/193	95	0	98	82
Cho WC [15]	0.109 ± 0.033	1.872 ± 0.212	NA	NA	NA	NA	NA	NA	NA	NA	NA
	17.06 ± 2.55	710.77 ± 250.42									
Sung HJ [4]	13.89 ± 37.18	190.49 ± 234.70	190.49 ± 134.70	302.76 ± 305.21	116.38 ± 81.13	7/140	90/170	90	7	80	133
Dowling P [18]	9.8	NA	141.6	54.8	172.2	NA	NA	NA	NA	NA	NA
Kanoh Y [9]	8.2 ± 10.55	158 ± 448.7	NA	NA	NA	0/13	24/24	24	0	0	13

N, cases; NA, unavailable; true positive; *LAC* lung adenocarcinoma; *LSCC* lung squamous cell carcinoma; *SCLC* small cell lung cancer; *FP* false positive; *FN* false negative; *TN* true negative

### Comparison of SAA level in different histological classification of lung cancer

**Fig. 3:**
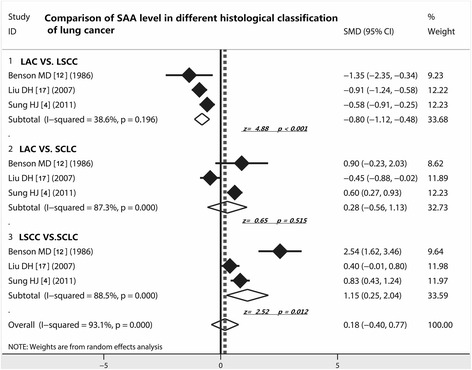
Comparison of SAA level in different histological classification of lung cancer. SAA level was higher in LSCC than in LAC and SCLC, demonstrating that SAA may specially play a significant role in LSCC; LAC, lung adenocarcinoma; LSCC, lung squamous cell carcinoma; SCLC, small cell lung cancer; CI, confidence interval

As shown in Table 3, three studies [4, 12, 17] compared the SAA level between LAC and LSCC. The random-effect combined SMD was −0.80 (95 % CI −1.12 to −0.48; *Z* = 4.88, *P* < 0.001), indicating SAA level was higher in LSCC than in LAC. Comparing LAC with SCLC, the random-effect combined SMD was 0.28 (95 % CI −0.56 to 1.13; *Z* = 0.65, *P* = 0.515), indicating no difference was confirmed. However, the random-effect combined SMD that resulted from the comparison between LSCC and SCLC was 1.15 (95 % CI 0.25 to 2.04; *Z* = 2.52, *P* = 0.012), demonstrating that SAA level in LSCC was higher than in SCLC. To conclude, the LSCC displayed the highest SAA level among the three histological type of lung cancer (LAC, LSCC and SCLC), implying that SAA may be biomarker of LSCC especially (Fig. 3).

### Analysis of sensitivity and publication bias

**Fig. 4:**
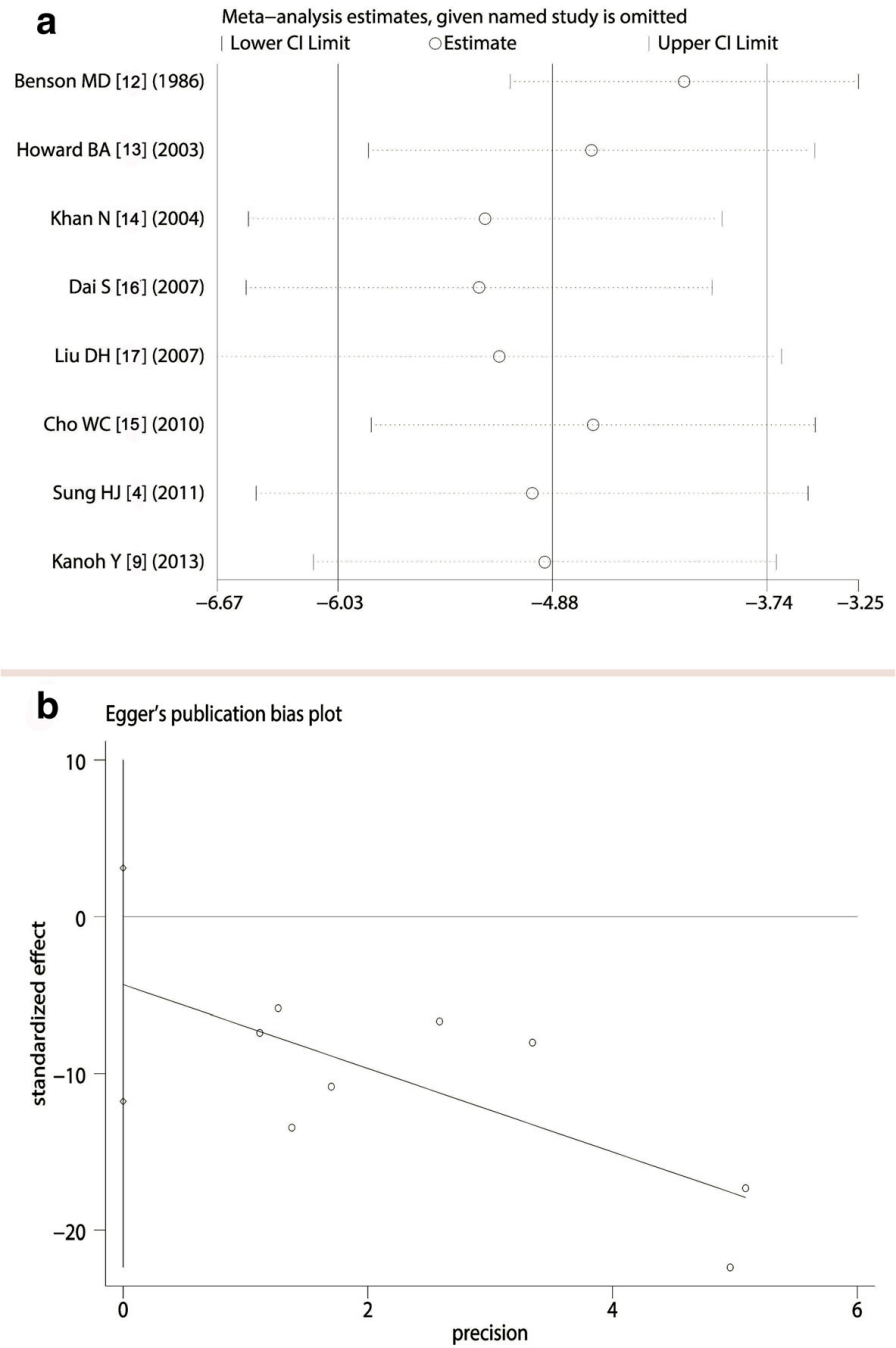
Analysis of sensitivity and publication bias. **a** For comparison of SAA level between lung cancer and healthy individuals, exclusion of studies on an individual basis did not substantially modify the estimators; **b** Z value of the Egger test was −1.42 (Pr > |z| = 0.25), implied that there was no publication bias for these studies

The sensitivity analysis showed that the exclusion of studies on an individual basis did not substantially modify the estimators and affect the final statistical efficacy, with a SMD pool oscillating between −2.40 and −9.76 (Fig. 4a). We employed the Egger test and Begg’s Test to adjudge whether there was a publication bias or not. The results showed that Z value of the Egger test was −1.42 (Pr > |*Z*| = 0.25), and T value of Begg’s Test was −0.99 (P > |t| = 0.386). With the fact that, no publication bias was considered (Fig. 4b).

### Sensitivity and specificity of SAA for distinguishing lung cancer

**Fig. 5:**
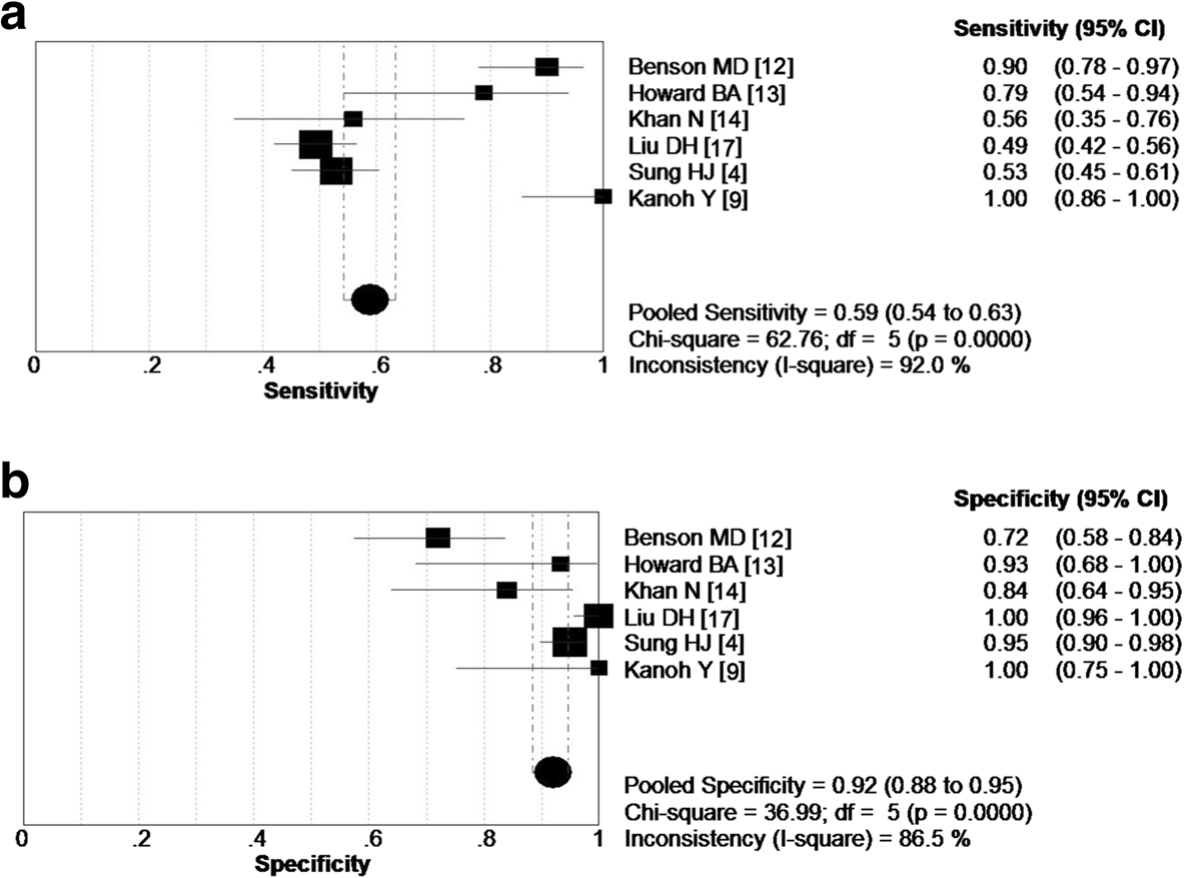
Sensitivity and specificity of SAA for the diagnosis of lung cancer. **a** Pooled sensitivity was 0.59; 95 % CI was 0.54 to 0.63; **b** pooled specificity was 0.92, which suggested that SAA has a relatively higher specificity; CI, confidence interval

As shown in the forest plot of the sensitivity (Fig. 5a), the sensitivity of SAA in included studies ranged from 0.53 to one (pooled sensitivity = 0.59; 95 % confidence interval = 0.54 to 0.63). However, the pooled specificity of SAA for distinguishing lung cancer reached up to 0.92 (95 % CI, 0.88 to 0.95) (ranged from 0.72 to one), which demonstrated that increased SAA had higher specificity in discerning lung cancer (Fig. 5b).

### Diagnostic accuracy of SAA for discerning lung cancer

**Fig. 6:**
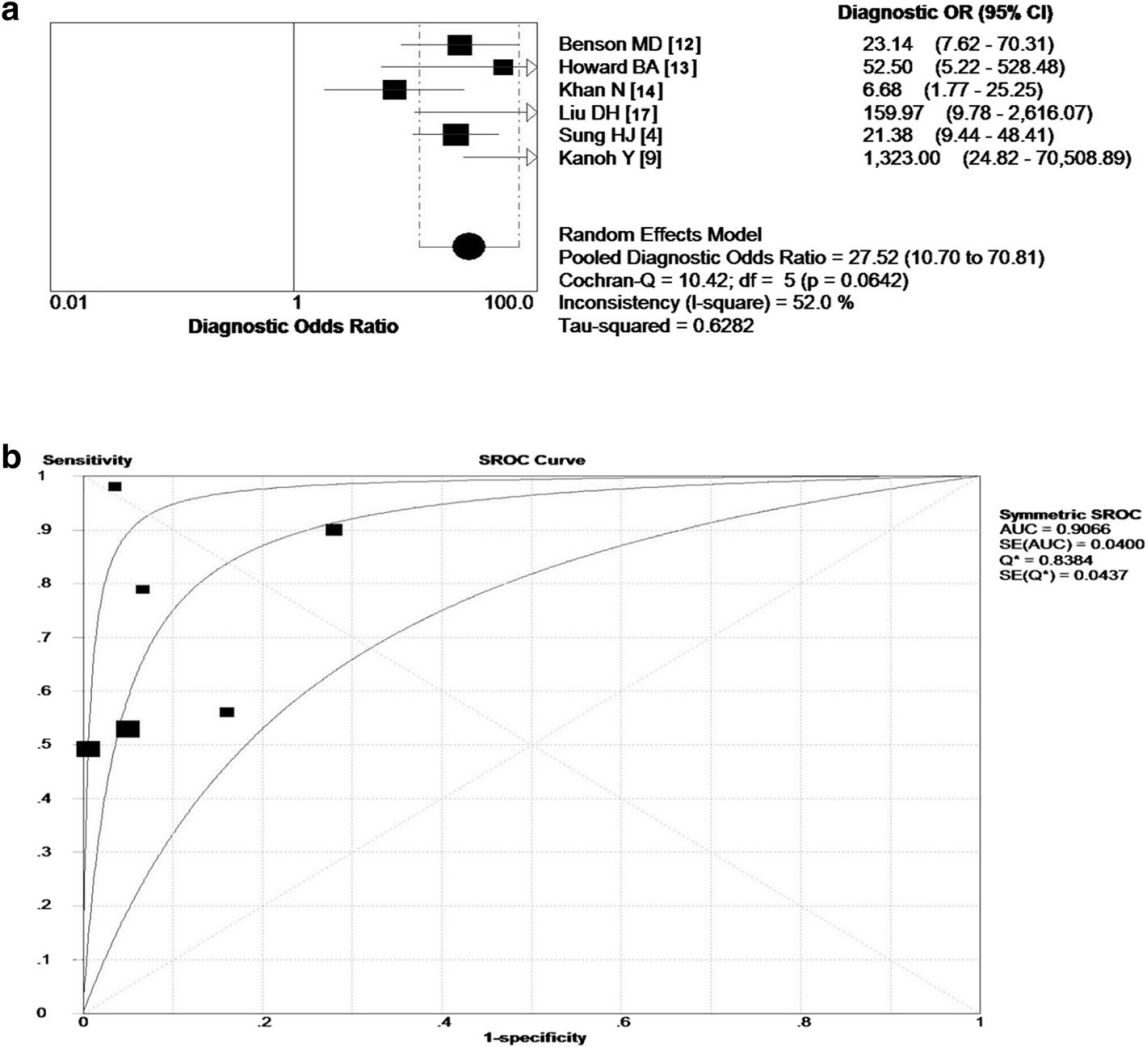
Diagnostic accuracy of SAA to lung cancer. **a** The overall Diagnostic Odds Ratio (DOR) of included studies were 27.52 (*p* = 0642), which indicated that SAA had a ability to discern lung cancer; **b** The balanced point for sensitivity and specificity (the Q‑value) was 0.8384. The area under the curve (AUC) was 0.9066, indicating that the overall accuracy was impressive. SORC, summary receiver operating characteristic; OR, odds ratios

The overall diagnostic odds ratio (DOR) of included studies were 27.52 (*P* = 0.0642), with the scope ranged from 6.68 to 1323 in these studies (Fig. 6a). Figure 6b summarized the test performance of each study by using the SROC curve, and the balanced point for sensitivity and specificity (the Q‑value) was 0.8384. The area under the curve (AUC) was 0.9066, indicating that the overall accuracy was impressive.

## Discussion

Now, lung cancer has become the leading cause of malignancy-related deaths in the world [17, 19], the 5-year survival rate for lung cancer is only slightly better than 10 %. Lung cancer exhibits the highest mortality of all cancers mainly because most patients have developed into the advanced stage when the diagnosis of disease is confirmed [4]. People believed that stable biomarkers which can be routinely measured in easily accessible samples effectively help make early-stage diagnosis for lung cancer [20]. Blood is an easily accessible and rich body fluid. Research shows that blood plasma and serum contain specific proteins that provide potential circulating biomarkers [21]. For example, the level of acute-phase SAA often increases in cancer patients, even at its early stage. This fact was registered in different common cancers, such as lung, ovarian, renal, uterine, and nasopharyngeal cancer and in melanoma [7].

In this study, we reviewed the relevant studies comparing the expression of SAA between lung cancer and healthy individuals and found that patients with lung cancer showed a higher SAA level than those of healthy group. This result indicated that a higher SAA level certainly correlated with occurrence and development of lung cancer and that SAA could be an indicator of lung cancer. We noticed that there was methodology heterogeneity that existed between included studies, but we found that included studies had a very good clinical homogeneity. For instance, no biases of age and diagnosis were observed in these studies. Moreover, patients included in these studies were from East Asia, Europe and America, which embodied the globalization and thus eliminated the ethnic bias. In order to strength the reliability of results, we made a comparison of SAA positive rate and showed that SAA positive rate of patients with lung cancer was higher than that of healthy individuals.

We also found that most of studies had a moderate to higher quality assessed by using the QUADAS-2 scoring system. Subsequent analysis of sensitivity further showed that the exclusion of studies on an individual basis did not substantially modify the overall effect of meta-analysis. Bias evaluation [11] in our analysis suggested that there was not a significant publication bias. Together, the results of this meta- analysis should be more stable. Previous studies have found that SAA can distinguish lung cancer patients from healthy controls as well as predict prognosis of lung cancer [8, 12, 15, 17]. SAA is secreted during the acute phase of inflammation, including invertebrates and vertebrates, suggests that SAA has an essential role in all animals including humans [15]. Study point out that overexpression of SAA is always correlated with inflammation and acute-phase responses [16]. Further, investigation on cancers reveals that chronic inflammation is associated with development and progression of malignant tumors, and inflammatory factors can be applied as diagnostic and prognostic indicators for some malignant tumors. SAA is a kind of inflammatory factor, adding our findings, thus showing that there is strong relationship between chronic inflammation and incidence of lung cancer.

It is likely that SAA in pulmonary inflammation may be temporarily elevated and recovered soon after the elimination of infection, but not the same in cancers, which may represent a primary difference between benign and malignant diseases of lung [17]. In our analysis, we were excited to find that LSCC displayed a much higher SAA level than LAC and SCLC, which gave us a very significant clue that we might specially use SAA for discerning LSCC from others. The results also confirmed by subsequent evidence that overexpression of SAA even was detected western blot analysis in LSCC, but not in others [4]. It is widely known that there has still no efficient biomarker for LSCC diagnosis so far. As an indicator of the potential usefulness of SAA in the diagnosis of lung cancer, in particular in LSCC, we ought to investigate deeply the role of SAA in LSCC in the future.

It is unassailable, as a diagnostic marker, a good sensitivity and specificity are very important. In this meta-analysis of diagnostic test we found that the increase of SAA has a higher specificity (0.92; 95 % CI: 0.88-0.95) for discerning lung cancer. However, the pooled sensitivity was only 0.56 (95 % CI: 0.54-0.63), which suggested that SAA has a better role for distinguishing lung cancer but not for screening. Thus, when biopsy of tumor tissue is absent or insufficient in clinic, we may use the SAA as an indicator to discern lung cancer. However, the absence of increased SAA should not mean the impossibility of lung cancer. The DOR always indicate the test accuracy of a biomarker that bind the compromise of sensitivity and specificity to a quantitative data. People believed that a higher DOR values suggest a higher accuracy of diagnosis. In our analysis, the pooled DOR was 27.52, supporting that the SAA assay could be advantageous in the diagnosis of lung cancer. The definitive diagnosis of lung cancer usually requires tissue biopsies of adequate size. However, sometimes the tissues for pathology biopsy were insufficient, and then a test with SAA would help improve the differential diagnosis. The SROC curve has been recommended to represent the performance of a diagnostic test [11]. Our analysis showed that the AUC of SAA was 0.9066, which indicated that the SAA has good value in terms of the discerning diagnosis of lung cancer. From the present data, we think that every patient with suspected lung cancer should undergo the test of SAA. Patients with positive SAA level should undergo further invasive procedures biopsies, and produce a final diagnosis.

The limitations of this study are as follows: first, some studies had small size; second, some studies had relatively low quality in clinical and statistical designs; third, detection methods of SAA were different in these studies. In the future, it is very crucial to compare the SAA status in different histology classification of lung cancer with large samples, multiple clinical centers. Although some deficiencies existed, the study still drew a conclusion that the SAA assay could be advantageous in the diagnosis of lung cancer, especially for LSCC.

## Conclusions

Patients with lung cancer showed a higher SAA level than those of healthy individuals, suggesting that increased SAA correlated with the occurrence and development of lung cancer. In addition, the fact, SAA has a relatively higher specificity, suggested that SAA could be a new biomarker for discerning lung cancer, especially for LSCC.

## Abbreviations

AUC: Area under the SROC curve
CI: Confidence interval
DOR: Diagnostic odds ratio
LAC: Lung adenocarcinoma
LSCC: Lung squamous cell carcinoma
M ± SD: Mean ± standard deviation
OR: Odds ratio
QUADAS: Quality assessment of diagnostic accuracy studies
SAA: Serum amyloid A
SROC: Summary receiver operating characteristic curve
TNM: Tumor node metastases
